# Salbutamol transport and deposition in healthy cat airways under different breathing conditions and particle sizes

**DOI:** 10.3389/fvets.2023.1176757

**Published:** 2023-07-18

**Authors:** Rocio Fernández-Parra, Pascaline Pey, Carol Reinero, Mauro Malvè

**Affiliations:** ^1^Department of Small Animal Medicine and Surgery, Faculty of Veterinary Medicine, Universidad Católica de Valencia San Vicente Mártir, Valencia, Spain; ^2^Antech Imaging Services, Irvine, CA, United States; ^3^Department of Veterinary Medicine and Surgery, University of Missouri, Columbia, MO, United States; ^4^Department of Engineering, Public University of Navarre (UPNA), Pamplona, Spain; ^5^Biomedical Research Networking Center in Bioengineering, Biomaterials and Nanomedicine (CIBER-BBN), Madrid, Spain

**Keywords:** computational fluid dynamics (CFD), lower airway disease, feline, bronchospasm, inhalation therapy

## Abstract

Salbutamol is a bronchodilatator commonly used for the treatment of feline inflammatory lower airway disease, including asthma or acute bronchospasm. As in humans, a pressurized metered dose inhaler (pMDI) is used in conjunction with a spacer and a spherical mask to facilitate salbutamol administration. However, efficacy of inhalation therapy is influenced by different factors including the non-cooperative character of cats. In this study, the goal was to use computational fluid dynamics (CFD) to analyze the impact of breathing patterns and salbutamol particle size on overall drug transport and deposition using a specific spherical mask and spacer designed for cats. A model incorporating three-dimensional cat airway geometry, a commercially available spherical mask, and a 10 cm spacer, was used for CFD analysis. Two peak inspiratory flows were tested: 30 mL/s and 126 mL/s. Simulations were performed with 30s breathing different inspiratory and expiratory times, respiratory frequencies and peaks. Droplet spray transport and deposition were simulated with different particle sizes typical of the drug delivery therapies (1, 5, 10, and 15 μm). The percentage of particle deposition into the device and upper airways decreased with increasing particle diameter during both flows imposed in this cat model. During increased mean ventilatory rate (MVR) conditions, most of the salbutamol was lost in the upper airways. And during decreased MVR conditions, most of the particles remained in suspension (still in hold-up) between the mask and the carina, indicating the need for more than 30 s to be transported. In both flows the percentage of particles traveling to the lung was low at 1.5%–2.3%. In conclusion, in contrast to what has been described in the human literature, the results from this feline model suggest that the percentage of particles deposited on the upper airway decreases with increasing particle diameter.

## 1. Introduction

Inhalation therapy at-home or at the hospital is commonly used in cats (1). The primary treatment of feline lower airway disease (FLAD) is glucocorticoids with or without bronchodilators (1–3). Oral medication is generally considered to be effective and affordable, but some cats are challenging to medicate by this route and systemic side effects can be problematic (4). Parenteral treatment may be used during hospitalization but inhalational treatment has important advantages including direct application to the site of action (lower respiratory tract), fewer systemic adverse effects, and for certain inhaled bronchodilators, a more rapid onset of action (5). However, inhaled medications are not without their own set of limitations. In a recent online survey study (4) difficulties in administration of glucocorticoid or bronchodilator inhalant therapies were reported in 28% and 31% of owners, respectively. In another study of FLAD in which cats received metered dose inhalant budesonide, 55.8% of owners withdrew therapy due to high cost, lack of time for twice daily treatment, and/or drug ineffectiveness (6). Nebulization of liquid medications has been proposed as an alternative treatment (7, 8), but further studies are needed to test the efficiency of drug delivery with this technique. Furthermore, nebulizers require good hygiene to avoid contamination and some nebulizers are noisy and bulky (9).

In the case of feline asthma, one type of inflammatory FLAD, airways have increased responsiveness to inhaled aeroallergens or irritants provoking bronchoconstriction (10). Clinically, tachypnoea, wheezing, coughing or labored respiration (typically with increased expiratory effort) are observed, which can be treated with bronchodilators (2, 11) such as salbutamol. Clinically, bronchodilators may also be used as a preventive therapy against bronchospasm during bronchoscopy and bronchoalveolar lavage (12) or as an emergency drug during bronchospasm through general anesthesia. Inhaled salbutamol, also known as albuterol, is marketed as a pressurized metered dose inhaler (pMDI) with doses ranging between 25 to 100 μg per actuation (13). In general, the pMDIs are filled with the pressurized drug in the form of either a solution or suspension of drug particles in the micron size ranging between 1 to 100 μm and dispersed within a suitable propellant in its interior (14). The diameter of salbutamol pMDI particles are in the range of microns (15, 16). Most computational studies use particle sizes ranging between 1 and 17 μm to test inhaled particle deposition (17, 18). The transport and distribution of particles are mainly affected by their size and the flow patterns during inhalation in humans (16). But the velocity, relative humidity (19), devices used to apply them, or the anatomy (20, 21) also play roles. Spacers are chamber extensions to the pMDI attached to a mask that allow the inhalation of the drug without synchronization with the actuation of the inhaler (22). However, their efficiency is controversial (23). In a previous study using computational fluid dynamics (CFD), we demonstrated how the choice of different devices to administer salbutamol in cats with pMDIs impact the amount of drug that ultimately reaches the lung (24).

In human medicine, therapeutic effectiveness is strongly related to the correct use of devices and to the inhalation technique (25, 26). Additionally, the respiratory system has developed defense mechanisms against inhaled particles which must be overcome by inhalational therapies (27). In veterinary medicine, devices are not fully optimized to the individual patient or to the underlying pathological condition (28). Veterinarians are able to choose from a large number of commercial devices and masks designed for cats or adapted from humans. Unfortunately, masks are commercially available only in standard dimensions and clinically may not fully conform with a necessary tight seal to the muzzle of an individual cat. Furthermore, it is difficult to assess if the inhalation technique is well or poorly performed. Commands to initially exhale, fully inhale the medication, and breath hold as is done in humans, cannot be performed in cats and likely contributes to suboptimal drug deposition in the lungs (29). Furthermore, any unwillingness of cats to tolerate correct use of the device is a major limitation to its clinical use. Clinically, we have observed that inhalation therapies where restrain of the cat is necessary can be even more challenging leading to drug waste or increased stress of the cat. Acute disease exacerbation may modify the respiratory rate and pattern. There are also scenarios (e.g., critically ill hospitalized cats) where hypoventilation is more common. The deposition of particles of these scenarios is unknown.

The size of aerosolized particles during therapeutic delivery is essential to determine location and amount of drug deposition in the airways (30). The transport mechanism depends on particle diameter, with impaction being the main phenomenon driving micro-particles and diffusion driving nanoparticles. In the human airways, while deposition is small for particles less than 1 μm, it tends to increase in the range of micron (16, 31, 32). Additionally, breathing conditions affect the transport and the deposition of particles in the airways with higher flow rates tending to increase deposition for larger particle sizes (31). In animals, limited studies in rabbits, rats, monkeys and mice have investigated transport and deposition of aerosolized particles in the nasal and upper airways (33–38). Specifically in cats, data on the behavior of different aerosolized particle sizes with different peak inspiratory flows has yet to be reported.

Our previous work was a proof of concept using computed tomographic images based CFD model of upper airways from a single cat, using reasonable inspiratory and expiratory peak flow with a fixed salbutamol particle diameter of 10 μm and different devices. In the present study we have considered two ventilation conditions corresponding to decreased and increased mean ventilatory rate (MVR) or, in other words, minute ventilation that match better with the clinical situation of the animal when inhalant therapy is carried out during hospitalization (critical/sedated or stressed, respectively). Moreover, we have considered and studied the deposition and transport of different salbutamol particle sizes typical of drug delivery with a single device (10 cm length spacer and spherical mask).

The aim of this study was to use CFD to evaluate the distribution of microparticles according to two flows and particle size of salbutamol using a single device and anatomical healthy feline model. We hypothesized that larger particle sizes and increased MVR would enhance particle waste, while decreased MVR would enhance delivery of smaller particles to the lung.

## 2. Materials and methods

### 2.1. Animal model and computed tomographic (CT) images

A previous geometrical reconstruction of a CT image from a client-owned domestic shorthair cat of 3 years and 8 months of age that presented to our hospital for otitis media with no other concomitant respiratory or systemic disease, was used for further simulations. The cat weighed 4.25 kg, was sedated, breathing spontaneously, and placed in sternal recumbency with the head elevated and neck fully extended for a head to thorax CT. The use of images was approved by the Clinical Research Ethical Committee of the École Nationale Vétérinaire d’Alfort (ENVA), France (number: 2020-05-30).

Non-contrast-enhanced multidetector computed tomography (MDCT) examination was carried out using a 64-detector-row CT system (Brilliance 64; Philips, Amsterdam, Netherlands). The CT scan was obtained using a matrix of 768 × 768, tube voltage of 120 kV, tube current of 196 mA and a display field of view of 35 cm and a pitch of 0.5. Images for the study were acquired from the nostrils to the most caudal border of the lungs. One-millimeter-thick images were reconstructed using a high-resolution algorithm. The scan was reviewed using a commercial medical imaging software, DICOM (Digital Imaging and Communications in Medicine) viewer (Horos v.1.1.7., 64-bit, HorosTM, US) using a lung window (window width (WW): 1600; window level (WL): -550).

### 2.2. Geometrical reconstruction and numerical discretization

**Figure 1:**
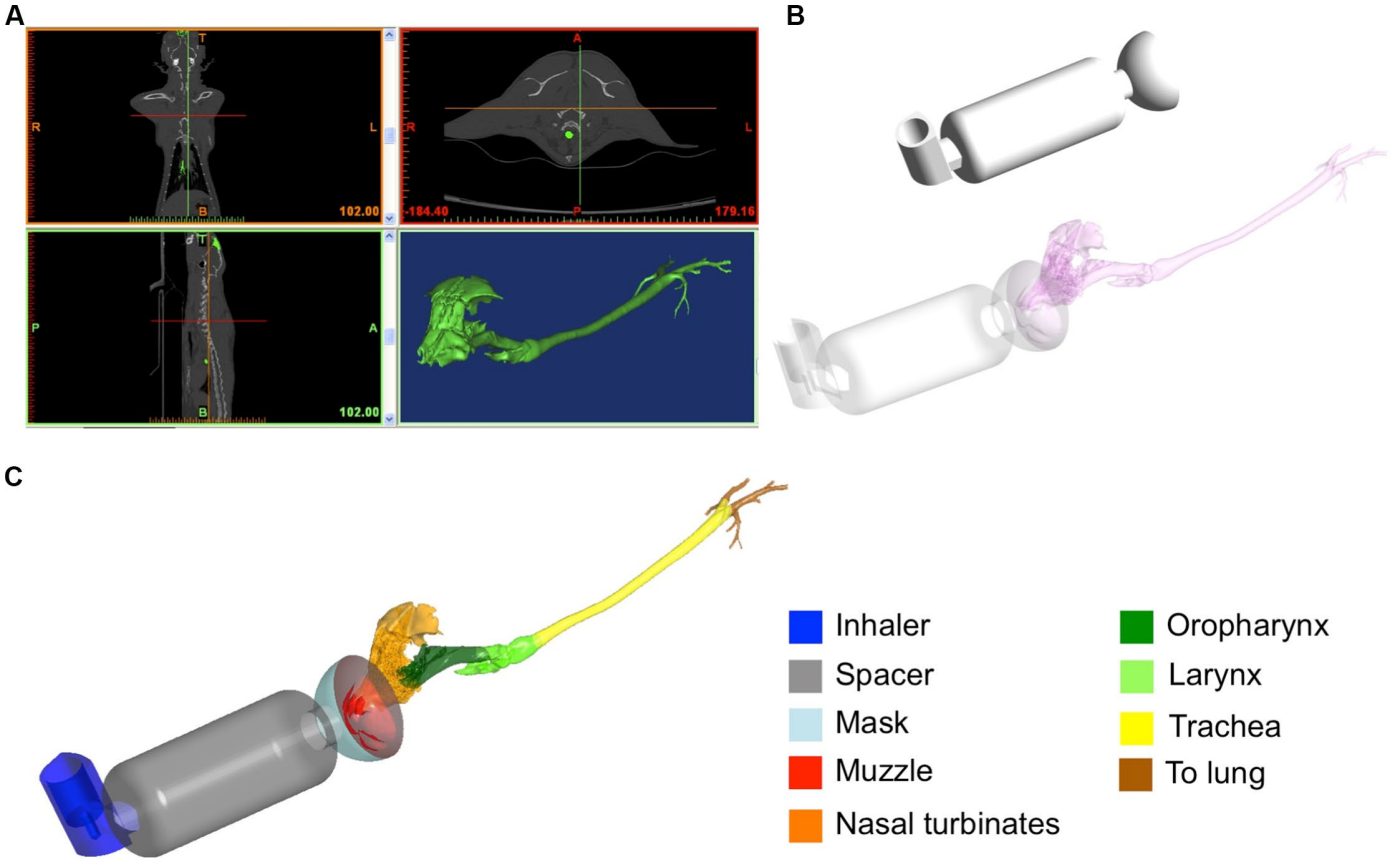
Reconstruction of the geometrical model starting from the CT-images of the cat **(A)**. Considered devices designed using CAD software: inhaler, spacer of 10 cm length and spherical pre-oxygenation mask **(B)**. Final geometrical domain considered for the simulations **(C)**.

The creation and discretization of the model has been extensively described and discussed in a previous study (24). Briefly, using the commercial software MIMICS (Materialise Software, Leuven, Belgium), using the CT images of the cat, a three-dimensional geometry of the upper airways including nasal cavity, naso-pharynx, oropharynx, larynx, and trachea was created (see Figure 1A). The reconstruction was performed manually. The geometry was exported in STL (Stereo Lithography) file and successively imported in the meshing program Ansys IcemCFD of the commercial package Ansys (v.20, Ansys Inc.). The devices (inhaler; Ventolin Evohaler 100 micrograms GlaxoSmithKline, Brentford, UK, spherical mask and 10 cm length spacer without valve; Aerokat^™^, Trudell Animal Health, ON, Canada) were created in the software package Rhinoceros (Robert McNeel & Associates, Seattle, WA, United States) (Figure 1B) and added to the cat STL file using Boolean union tools and functions. The final geometry is depicted in the Figure 1C. An automatic discretization of the geometry consisting of filling in the volume (composed by all the imported regions) with tetrahedral elements and the surfaces with triangles was carried out. As widely known in computational biomechanics, numerical models comprised of a small number of elements permits short computational costs but provides low precision of the results. On the contrary, models with a large number of discretization elements implies high computational costs with more precise results. For these reasons, prior to the final simulations we performed a mesh independence study to assess the dependency of the results on the number of elements composing the computational geometrical volume. This analysis was described previously (24) and demonstrated that greater than 23.5 million elements provides only higher computational costs without improving the precision of the solution. As a consequence, a grid composed of approximately 23.5 million elements was ultimately chosen. This mesh consisted of 2.5 million elements for the pre-oxygenation mask, 3 million elements for the spacer, and 18 million elements for the cat airways.

### 2.3. Computational analysis

The software package divided the simulations in two main parts: the continuous phase, consisting of the flow entering and going out to the computational domain, and the discrete phase consisting of a finite number of particles traveling within the domain and subjected to a specific behavior depending on the continuous phase (flow structure) and on collision with the airways.

#### 2.3.1. CFD modeling (continuous phase)

The mesh obtained after the volume discretization of the cat was imported into a simulation software package (Ansys CFX, v.22, Ansys Inc., Canonsburg, PA, United States).

The governing equations of motion of the gas flow used were the Reynolds averaged Navier-Stokes equations:


$$\nabla .\bar{V}=0$$

$$\frac{\partial \bar{V}}{\partial t}+\nabla .\left(\bar{V}\times \bar{V}\right)=-\frac{\nabla p^{\prime }}{\rho }+\left(\nabla .\nu_{eff}\;\left(\nabla \bar{V}+{\left(\nabla \bar{V}\right)}^{T}\right)\right)$$

where $\bar{V}$ is the averaged fluid velocity vector, p′ is modified pressure, ρ is the gas density, $\nu_{eff}$ is the effective kinematic viscosity and $\frac{\partial \bar{V}}{\partial t}$ is its partial derivative of the velocity $\bar{V}\;$respect to the time *t*, ∇ is the nabla symbol, $\nabla \bar{V}$ is the gradient of the average fluid velocity and ${\left(\nabla \bar{V}\right)}^{T}$ its transposed. The latter can be obtained as sum of the gas kinematic viscosity ν, and the turbulence kinematic viscosity $\nu_{t}$:


$$\nu_{eff}=\nu +\nu_{t.}$$

For modeling the turbulence, the Wilcox *k* – *ω* model was selected. This model predicts the turbulence using a partial differential equation for the turbulence kinetic energy *k* and a partial differential equation for its rate of dissipation *ω* (39). The general form of the gas dynamics equations, the turbulence equations, the precise definition of all parameters and variables, and the discretization and resolution methods are found in the Ansys manufacturer’s manual (40).

The governing equations of the fluid mechanics were recursively solved by this software using the finite volume method applied to the computational domains, i.e., the generated mesh and using boundary conditions such as flow velocity or pressure among others. The exact mathematical formulation and the solving algorithms used by Ansys CFX are given in the software manual (40).

**Figure 2:**
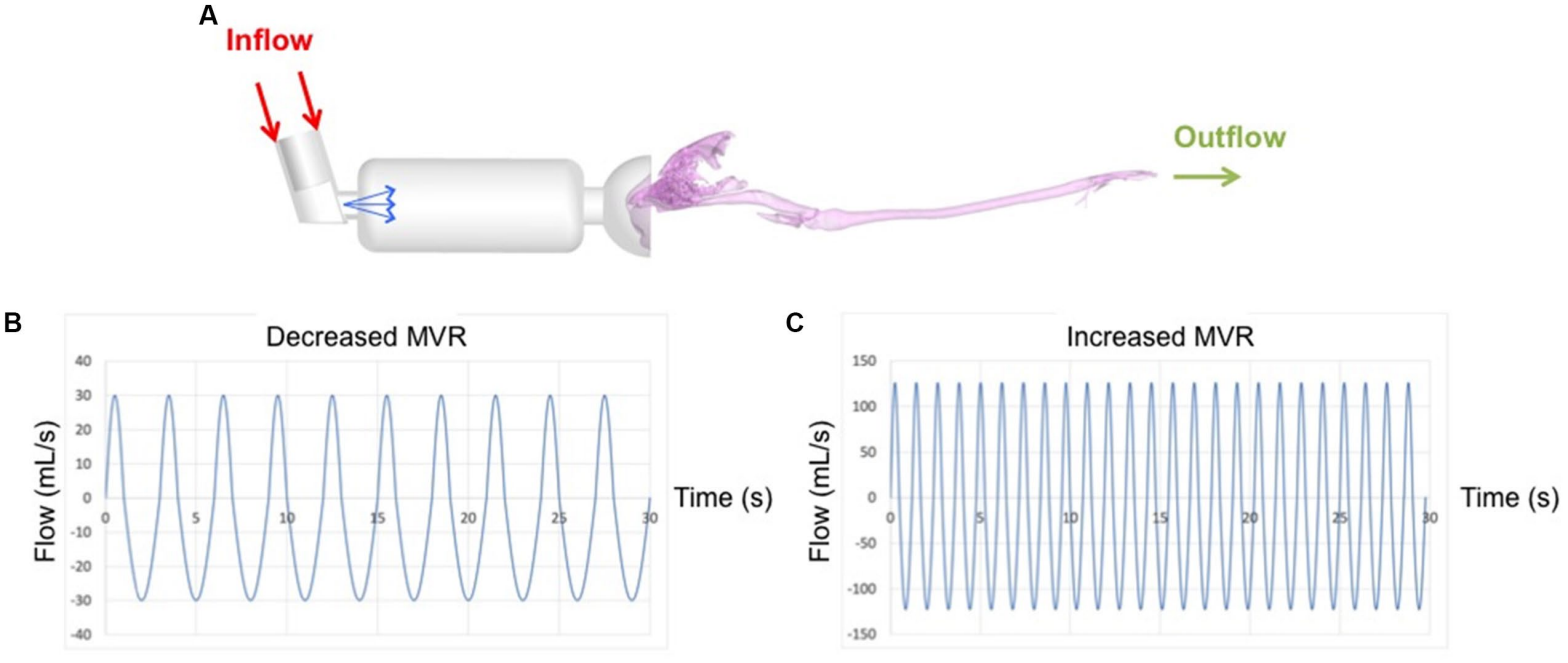
Complete computational model with boundary conditions used for the simulations. Red arrows represent location where the flow enters; green arrows represent the location where the flow exits. Blue arrows show the salbutamol spray which entrance velocity was 150 m/s **(A)**. Waveforms of 30s duration applied at the inlet and corresponding to 30 mL/s **(B)** and 126 mL/s **(C)** flows are plotted in the lower part of the figure.

The peak inspiratory flow under conditions of decreased MVR was 30 mL/s and under increased MVR was 126 mL/s (41). The 30 mL/s flow was obtained by means of a variable orifice flow sensor (GE Aestiva 5 Anesthesia Machine – Datex Ohmeda, Madison, WI, United States) in cats under general anesthesia. The peak inspiratory flow was imposed at the top of the pMDI (see Figure 2). As commented before, the flow was considered unsteady and turbulent with an initial turbulence intensity value of 5% (24).

A respiratory cycle of 3 s (1 s inspiration, 2 s expiration) and 10 respiratory cycles were used for the simulation under conditions of decreased MVR. Secondly, a respiratory cycle of 1.19 s (0.48 s inspiration, 0.71 s expiration) and 25 respiratory cycles were used for the increased MVR model (41). Both simulations had a duration of 30 s, divided into 3,000 time steps using a time step size of 0.01 s. The simulations adopted an air density of 1.185 kg/m^3^ and a viscosity of 1.83 10^−5^ Pa·s (42).

#### 2.3.2. Particle modeling (discrete phase)

The particle modeling was also computed in Ansy CFX and performed simultaneously with the fluid dynamics simulations. The software uses the discrete phase modeling (DPM) to obtain the trajectories and the fate of the particles injected through the pMDI nozzle. After defining the initial position, velocity and size, each particle is individually followed through the computational model using the force balance and local continuous phase conditions. Ansys CFX computes particle trajectories using Newton’s second law considering the drag force and gravity. This equation can be written as:


$$\frac{m_{p}dV}{dt}=\frac{1}{8}\rho d_{p}{}^{2}\;c_{D}{}_{p}\left|V-V_{p}\right|\left(V-V_{p}\right)+g$$

where ***V*** is the gas velocity, *d**V***/*dt* is its derivative respect to the time *t, **V**_p_* is the particle velocity, *m_p_* is the particle mass, ρ is the gas density, ***g*** is the gravitational force, *d_p_* the particle diameter and *c_Dp_* is the drag coefficient that is computed through the relation:


$$c_{Dp}=\frac{c_{D}}{c_{S}}$$

where *c_S_* is a correction factor of the drag coefficient *c_D_* that takes into account the irregularity of the droplet with respect to a spherical shape and it is function of the particle Reynolds number (Re_p_)


Rep=ρ|V−Vp|Vpμ

where μ is the dynamic viscosity of the gas.

The model included 2000 particles that are representative of a dose of 100 𝜇g per actuation (13). Particles are tracked through the fluid domain until one of the following three specific conditions happens: they impact and are trapped on the airways walls (deposition), they escape from the domain through outlets, or they continue in suspension in the flow (they could subsequently be exhaled or impact an airway wall in further breathings). If they escape through the outlets, they are automatically considered as traveling to the lungs. Particles are considered perfectly spherical and dilute and it is assumed that while flow influences the particle trajectories, the particles cannot interfere with the fluid motion (i.e., the continuous phase is not affected by the discrete phase). This hypothesis (also called “one way coupling”) is applicable, as the considered particles sizes and the volume fraction are sufficiently small (32). In order to obtain a regional particle deposition, the model was divided into zones, as shown in the Figure 1C. In particular, we specified the device that includes the inhaler, spacer and mask, the muzzle and the upper airways, divided into nasal turbinates that also includes the sinus, oropharynx, larynx, trachea that includes the carina and primary bronchi. Particles getting into the bronchi or exiting through the outlet where considered as traveling “to lung”.

Also, for the particle modeling, prior to the simulation a particle number independence study was carried out to find out the necessary number of particles to be injected in the fluid domain, i.e., to prove if 2000 particles are representative of a dosage of 100 per actuation. A sensibility analysis with different particle numbers was performed in a previous study (24) and concluded that 2000 particles are effectively representative of the considered actuation dose. The initial droplet diameters considered in this study were 1, 5, 10, and 15 𝜇m, based on previous studies (15, 17, 18). The initial spray velocity was 150 m/s with a cone angle of 15 (43). Furthermore, the pMDI actuation was applied prior to the first breathing cycle in order to simulate clinical conditions.

The salbutamol spray was modelled in CFX using a specific available model called “gas–liquid interactive Enhanced Taylor Analogy Breakup (ETAB) model” (13). This model predicts the formation of droplets caused by the drag force due to the interaction between gas and particle and their different velocities. The droplets formation takes place accordingly to the ratio between the inertia force to surface tension, also called Weber number (We), that combines the droplet deformation and the liquid surface tension as follows:


$$We=\rho \frac{V^{2}\;r}{\sigma_{L}}$$

where *ρ* is the gas density, *V* is the relative velocity between gas and particle, *r* is the particle radius, and *σ_L_* is the coefficient of liquid surface tension.

The pMDI used in the model consisted of a canister connected to a metering valve capable of producing variable dosages. As commented before, in this study we used 100 μg (13) delivered through an actuator-nozzle of 0.5 mm diameter located at the center line of the canister in a centered position with respect to the mask (Figure 2).

The numerical models allow computation of flow velocity and structures inside the cat airways and devices. The structure of the flow is depicted using 3D streamlines while the flow intensity is represented using a heatmap.

All computations were performed on an Intel Core i9 workstation with 2.3 GHz frequency, 32 GB RAM and parallelized on 8 processors. The computational cost of the simulations under decreased MVR was 254.5 h while during increased MVR condition was 273.2 h.

#### 2.3.3. Validation of the model

The computational model used in the present study was validated in a previous work (24). Briefly, the particle transport and deposition were validated using idealized human airways (including the oral tract through the first 4 bifurcations), classical breathing conditions (15, 30, and 60 L/min) and a particle diameter of 10 μm. In this model, particles were uniformly distributed to the oral entrance and released directly in the oral cavity without using the inhaler. Aerosol delivery was evaluated by means of steady simulations using the aforementioned breathing conditions and turbulent flow. Deposition fractions within the oral tract and the main bifurcations were computed and systematically compared with the results of Zhang and co-workers in 2005 (32). The obtained results confirmed the validity of the model, as the deposition fractions obtained with the present model were the same as those found by Zhang and co-workers (32).

Furthermore, because the spray modeling used in this work is based on those published in the literature (30, 43), using human airways without bifurcations, we have compared the spray performances obtained in the present model with the values obtained in those studies. It was clearly seen that the spray characteristics comply with the necessary requirements as the obtained results match well the findings previously published by Kleinstreuer and co-workers in 2007 (13) and by Yousefi and co-workers (43). For further details please see the paper from Fernandez-Parra et al. (24).

### 2.4. Data analysis

Airflow streamlines (peak inspiratory flow structures) and droplet deposition were represented using heat maps and described qualitatively for the various conditions in the three models. Percentages of particles deposited to the devices, to the upper airways and transported to the lung as previously defined for all conditions in each model were reported. Further data was analyzed with the statistical program IBM^®^ SPSS^®^ (Statistics software, version 30, Chicago, IL). A Shapiro-Wilk test was used to test for normal distribution of percentages of particles deposited to the aforementioned three regions and in suspension for further comparisons. Results are expressed in median (ranges) with maximum and minimum values. A Mann-Whitney U test was used to compare deposition percentages and a Kruskal Wallis test to compare differences in particle sizes between flow conditions. Values of *p* < 0.05 were considered statistically significant.

## 3. Results

### 3.1. Airflow patterns

**Figure 3:**
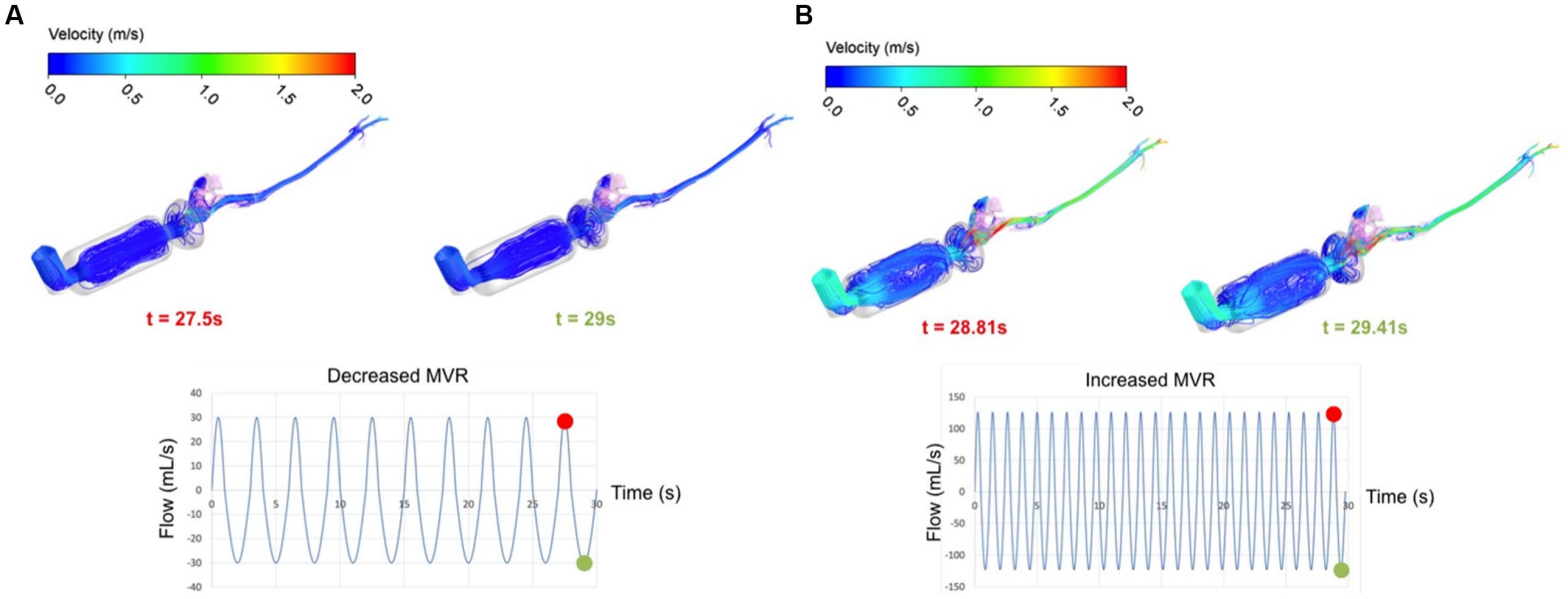
3D view of the flow structure colored using the velocity intensity for both breathing flows 30 mL/s **(A)** and 126 mL/s **(B)** at specified instants of time: at inhalation, *t* = 27.50s and 28.81 s, respectively (red circle on the curve) and at exhalation, *t* = 29.00s and 29.41 s, respectively (green circle on the curve).

The airflow structure within the cat airways is represented in Figure 3. Streamlines (lines indicating the direction of the flow) were colored with the velocity intensity for decreased and increased MVR during inhalation and exhalation in the last breathing cycle.

Comparing the two breathing conditions, there were obvious differences in intensity of the velocity, more marked during increased MVR (light blue in Figure 3B representing higher velocity in comparison with dark blue in Figure 3A). Additionally, there was a visible and consistent difference inside the spacer. A low breathing rate led to more ordinate and parallel streamlines in this device whereas for a higher breathing rate, the streamlines inside the spacer were more chaotic. These flow patterns likely influence salbutamol deposition as the particles are transported by airflow that presents changes in direction much more enhanced for 126 mL/s versus 30 mL/s.

High flow velocity is visible in both cases and for both inhalation and exhalation near the nasal region, where the flow entrance suddenly reduces from the spacer (during inhalation) and from the oropharynx (during exhalation). On the contrary, the laryngeal region shows lower velocity intensity compared to the nasal region. Although the larynx normally acts as a nozzle, decreased MVR seems to promote a uniform intensity through the larynx; under conditions of increased MVR there is only a slight high-speed region visible (see Figure 3).

**Figure 4:**
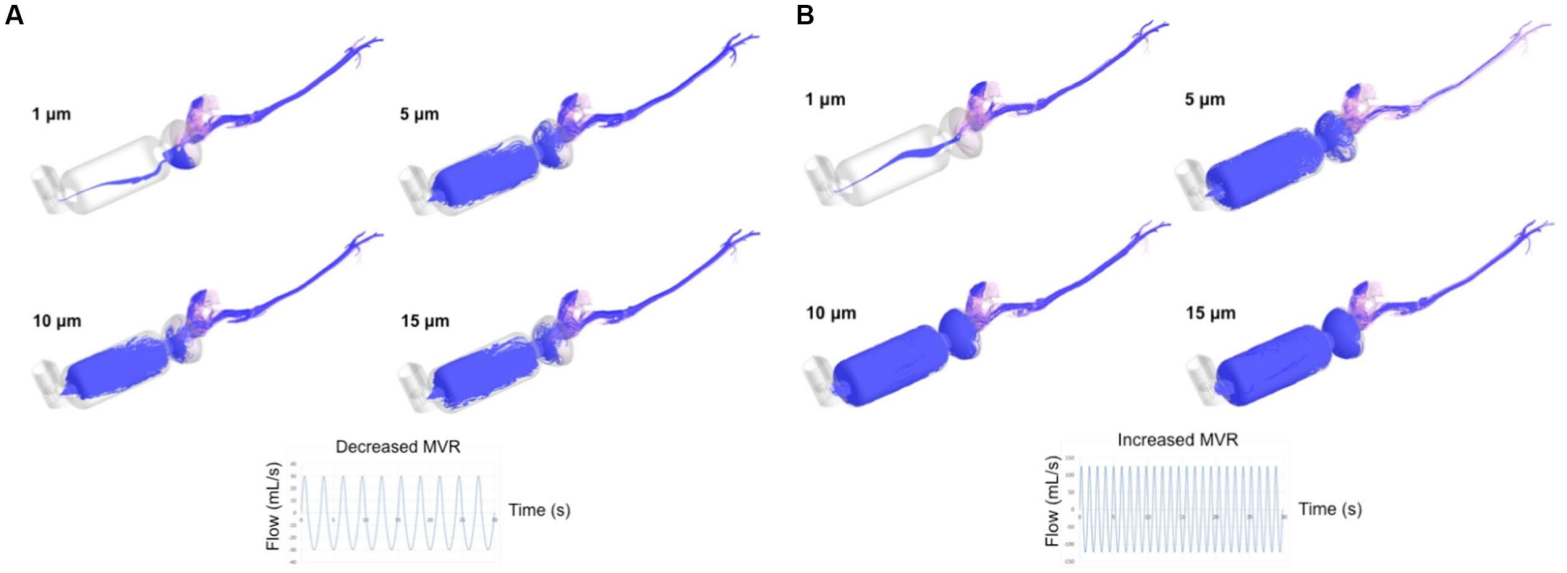
Time evolution of the salbutamol particles trajectories (depicted in purple) after 30s of both breathing flows, 30 mL/s **(A)** and 126 mL/s **(B)**. MVR: mean ventilatory rate. The trajectories represent the aerosolized salbutamol flow of particle injected through the MDPI orifice in the computational domain (devices + upper airways) and reaching the lung (model outlet).

Figure 4 shows the trajectories followed by particles of 1, 5, 10, and 15 μm during entire breathing cycles. The Figure depicts the aerosolized salbutamol flow of particles injected through the MDPI orifice and reaching the lung (the model outlet of the computational domain). A marked difference is visible in both ventilation conditions between particles of 1 μm with respect to the other sizes. One-μm particles are less influenced by the airflow inside the spacer compared to 5, 10, and 15 μm particles. Comparing the two flow rates for particles bigger than 1 μm, the trajectories follow the airflow structure in the spacer providing a higher number of particles in suspension for decreased MVR (compare with Figure 3A). Additionally, increased MVR leads to more chaotic trajectories in the spacer compared to decreased model (for example, compare the density of trajectories inside the spacer for particle >1 μm represented in Figures 4A,B).

### 3.2. Particle transport and deposition

**Figure 5:**
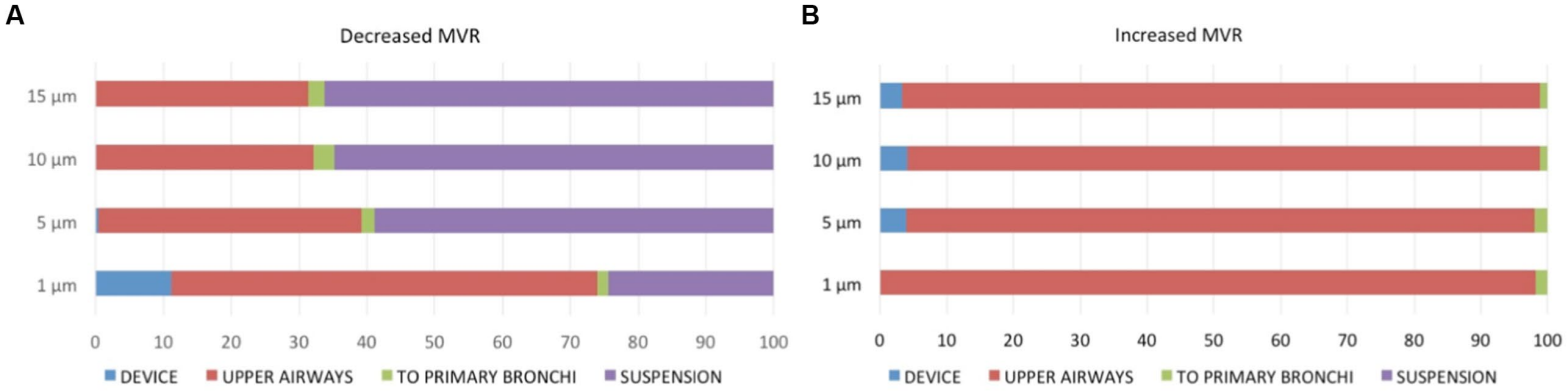
Percentage of particle distribution after 30 s of breathing flows of 30 mL/s **(A)** and 126 mL/s **(B)** into the three regions [device (blue), upper airways (red), to primary bronchi (green), as well as remaining in suspension (purple)].

All values in this section, deposition, reaching the lung and suspension refer to the entire cycles lasting 30s. The total percentage (%) of particles reaching the lung after 30 s of breathing flows of 30 mL/s (A) and 126 mL/s (B) are represented in Figure 5.

Particle deposition percentage was computed after 30s as ratio of deposited and injected particles times 100. These percentages of particles deposited at the device are summarized in Table 1. They represent the amount of drug, which is not able to reach the upper airways or the lungs.

**Table 1:** Percentage of particles of differing sizes deposited to the different parts of the devices after 30 s of breathing flows of 30 mL/s and 126 mL/s.

Flow	Particle size (μm)	Inhaler*	Spacer*	Mask*	Total*
Decreased MVR 30 mL/s	1	0	0	11.2	11.2
	5	0	0.3	0.1	0.4
	10	0	0	0.1	0.1
	15	0	0	0	0
Increased MVR 126 mL/s	1	0	0	0	0
	5	0.2	3.6	0.2	3.9
	10	0.2	4.0	0	4.2
	15	0.4	2.7	0.2	3.3

*All values are described in percentages (%). MVR: mean ventilatory rate.

Taking into account all particles sizes, similar particle waste due to deposition into the device was noted during decreased (11.7%) and increased (11.4%) MVRs. No particle deposition in any part of the device was noted during decreased MVR for particles of 15 μm size or during increased MVR for particles of 1 μm size.

Deposition of particles of different sizes deposited on the muzzle or in the upper airways (nasal turbines, oropharynx, larynx, and trachea) during 30 and 126 mL/s flows are represented in Table 2. Particle deposition in the nasal turbinates was at the highest value compared to other sites in both flows. During decreased MVR, the particle residue in this area was twice as great at a size of 1 μm compared to a size of 15 μm. Similarly, the amount of drug lost in the nasal turbinates during increased MVR was 1.5 as great at a size of 1 μm compared to a size of 15 μm.

**Table 2:** Percentage of particles of differing sizes deposited to the different areas of the muzzle or upper airways (defined as the nasal turbinates, oropharynx, larynx, and trachea including primary bronchi) after 30 s of breathing flows of 30 mL/s and 126 mL/s.

Flow	Particle size (μm)	Muzzle*	Nasal turbinates*	Oropharynx*	Larynx*	Trachea*	Total*
Decreased MVR 30 mL/s	1	1.45	54.8	2.9	2.4	1.1	62.5
	5	0.6	33.5	1.2	2.5	0.9	38.6
	10	0.4	28.2	1.2	1.4	0.9	32.0
	15	0.4	27.2	1.6	1.6	0.5	31.3
Increased MVR 126 mL/s	1	5.8	88.9	3.00	0.3	0.3	98.2
	5	6.7	82.5	2.5	1.35	0.8	93.8
	10	32.7	60.1	0.6	0.9	0.5	94.7
	15	35.2	58.7	0.7	0.8	0.1	95.5

*All values are described in percentages (%). MVR: mean ventilatory rate.

Medians and (range) of the percentage of particles deposited into the devices, upper airways, reaching the lungs (i.e., exiting the computational model) and in suspension during both flows are displayed in Table 3. No significant differences were observed between the particles traveling to the primary bronchi or those deposited into the device between both flows. During increased MVR, the deposition of particles in the upper airways was significantly higher compared to decreased MVR. Conversely, during 30 mL/s flow significantly more particles remained in suspension compared to the 126 mL/s flow.

**Table 3:** Percentage of total particle deposition into the device and upper airways or traveling to the lungs* and in suspension after 30 s of breathing flows of 30 mL/s and 126 mL/s.

Flow	Device	Upper airways	To the lung	Suspension
Decreased MVR 30 mL/s	0.25 (0.00–11.20)	35.45 (31.30–62.70)	2.3 (1.8–3.1)	61.78 (24.4–66.20)
Increased MVR 126 mL/s	3.58 (0.00–4.15)	95.13 (94.05–98.15)	1.53 (1.15–2.05)	0.00 (0.00–0.00)
Value of *p*	0.686	0.029	0.057	0.029

All values are described in percentages (%). The value of *p* was determined for comparisons between decreased and increased MVR for each location of particle deposition. *Lung refers to lower conducting airways distal to the primary bronchi to the parenchyma. MVR, mean ventilatory rate.

The percentage of particle deposition decreased with increasing particle diameter but there were no statistical differences between the distribution of particles into the device and upper airways (*p* = 0.869 and *p* = 0.801, respectively) or between those traveling to the lung or in suspension (*p* = 0.881 and *p* = 0.925, respectively) between different particle sizes.

## 4. Discussion

It is well established that the deposition of micro-particles tends to increase with increasing particle diameter, meaning there are wasted particles that will never reach the bronchi (32). In this study and contrary to what we hypothesized, the tendency observed for particle depositing in the upper airways walls decreased when the particle diameter increased, despite a lack of significant difference between location of deposition and particle size. Different to what is described in the human literature, particles of the largest size reached the bronchial area more easily and remained in suspension for a greater time than smaller ones (Figure 4). Airway bifurcations in the first few generations are subjected to high flow rates and hence, they may receive only small amounts of micro-particles due to largely amount of deposition that takes place upstream. At the flows studied, the percentage of particles traveling to the lung was low, ranging between 1.5%–2.3%. Inhalation in humans is performed through the mouth, with the oral cavity, oropharynx, larynx, and regions just upstream of the glottis being preferred locations for micro-particle deposition. In cats, inhalation is carried out mainly through the nose, and preferred locations for deposition of micro-particles of 10 μm are the muzzle and main airways independent of the type of device used for the therapy (spacer of 10 cm or 20 cm and conical or spherical mask) (24). Smaller particles (1–5um) tended to primarily deposit on the muzzle and in the nasal turbinates with far fewer reaching the lower airways; this pattern was exaggerated during increased MVR. We believe this is due to a more complex and narrowed feline upper airway anatomy compared to humans. We found a smaller amount of particle deposition into the devices in this study compared with the previous study (24). Using geometry derived from a single cat and a single particle diameter of 10 μm, we found a very low amount of drug traveling to the target, independent of the type of spacer or mask (24). However, in that study, different respiratory flow and frequencies were imposed (24).

Data of the percentage of inhaled salbutamol particle transport and deposition in cats is limited. A scintigraphy study proved an 86% positive rate of deposition into the lungs in 20 cats after nebulization of technetium Tc 99 m-diaminetriaminopentaacetic acid (99mTc-DTPA) into a spacer and fitting facemask (44). Pulmonary functional test evaluating bronchodilatation effectiveness of salbutamol administration has also been done (45). And the effectiveness of inhaled lidocaine in healthy and experimentally asthmatic cats was assessed by studying the flow, pressure and airway resistance and after preforming bronchoalveolar lavage fluid (BALF) analysis (8). Li and co-workers in 2007 demonstrated that deposition of micro-particle is due to impaction, secondary flow convection, and turbulent dispersion (46, 47). For this reason, in humans particles tend to deposit mainly at stagnation points (oral cavity, pharynx, larynx, and trachea) and rarely reach the deep bronchial region (21). In this study, deposition tends to be higher in the muzzle and in the nasal turbinates region due to its complex structure and the deposition was lower in the oropharynx and tracheal region. We also found that some micro-particles may travel and deposit in the sinus area, due to turbulent dispersion and secondary flow as described in rabbits (35). But in humans, the inhaled particles of 1 μm are effectively delivered distally (21), contrary to our results. In rodents, the maxilloturbinate region has been recognized as a very important and highly complex structure, accounting for over 50% airways resistance, bifurcating in several branches, and, in rabbits, acting as a filter for inhaled particles (35, 36). In this study, similarities of the nasal turbinates in cats may explain the large deposition in this region. Delivering therapy through an endotracheal tube (24) increases the percentage of particles traveling to the lung as the muzzle and maxilloturbinate region is bypassed.

Higher flow rates may modify the trajectory and distribution of smaller particles. There is a relatively large body of knowledge of aerosol particle behavior in human airways, including adults and children. In comparison, little is known in domestic animals. Previous studies have not focused on a veterinary clinical application such as the aim of improving efficacy of drug delivery for feline asthma. In the current study, during lowers flows, particles were deposited into nasal turbinates without reaching the oropharynx or remained in suspension after 10 breaths in 30 s. Cats should breathe through the mask and spacer for 7–10 s after each actuation (3). During periods of decreased MVR, it is clear the time needed is considerably longer. Lugo and Ballard in 2004 developed a human neonatal ventilator lung model and described a low albuterol delivery for 5–15 breaths and moderate increases in delivery for 30 breaths during 60s (48). In our study during conditions of decreased MVR, a high number of particles remained in suspension, indicating they have not deposited on any walls or reached the model outlets (meaning traveled to the lungs). In contrast, during increased MVR no particle remained in suspension over the same amount of time. This suggests that due to the low flow rate, a larger time and number of breaths would be necessary promote particle transport. The effect of breathing condition on the distribution and deposition of particles in humans has been standardized to three flow types; 30 L/min for normal, 15 L/min for light and 60 L/min heavy breathing patterns (15, 16, 30, 32). In this study, we have tried to simulate two different flows situations based in the previous literature (41, 49) and spirometry values from anesthetized cats. However, data reporting peak inspiratory flows in cats is scarce. With the respiratory conditions selected for this study, the percentage of salbutamol that travels to the lungs after one actuation during 30s is very low and is independent of the particle size. In human medicine, it is accepted that most inhalers only allow a small amount of the administered dose of drugs in the lungs. However, considering their efficacy and cost, this is not commonly considered problematic (27). As reported previously in a feline model of asthma, Leemans and co-workers in 2009, studied the antispasmodic effects of salbutamol pMDI and justified a single actuation in awake cats (45). Unfortunately, the necessary dose and breathing time in cats under different respiratory conditions is currently unknown.

The main limitations in this study include the assumptions of rigid, non-dynamic, and not deformable airways and the use of a single cat’s anatomy to model the airways which cannot take into account interindividual morphologic variability of the pet cat population. Thus, findings of this study may not be representative of the salbutamol inhalant delivery in all cats and clinical situations. However, this model is the first step to understand the influence of different flows and particles sizes in the inhalation therapies in cats. Additionally, the considered geometry did not take into account the entire lung, which would have been complex to image and reconstruct. Furthermore, additional ventilation conditions may be crucial for confirming the main findings of this study. Finally, in all the simulations, perfect fitting of the spherical mask on the cat muzzle has been assumed. However, it is clear that this is an ideal situation, and the unwilling patient may cause drug waste directly from the device. Studies using CT images-based models of asthmatic cats are left for future investigation.

In conclusion, different flow conditions modified particle transport in this model of device and cat airway anatomy. From a clinical point of view, during decreased MVR situations, longer breaths and times for medication delivery than typically recommended are needed. Results of this study suggest that increased MVR induced drug wastage. Further studies including different CT images-based models of asthmatic cats, varied cat anatomies, and different devices, drugs and dosages are necessary to further extend and assess the knowledge of drug delivering therapies.

## Data availability statement

The original contributions presented in the study are included in the article/supplementary material, further inquiries can be directed to the corresponding author.

## Ethics statement

The animal study was reviewed and approved by Clinical Research Ethical Committee of the École Nationale Vétérinaire d’Alfort (ENVA), France (number: 2020-05-30). Written informed consent was obtained from the owners for the participation of their animals in this study.

## Author contributions

RF-P and MM: conceptualization, methodology, formal analysis, investigation, data curation, and funding acquisition. MM: software. MM and PP: validation. RF-P, MM, and CR: writing—original draft preparation. RF-P, MM, PP, and CR: writing—review and editing and visualization. PP and CR: supervision and project administration. All authors contributed to the article and approved the submitted version.

## Funding

This study is supported by grants PID2021-125731OB-C31 and PID2021-125731OB-C33 from the Spanish Ministry of Science and Innovation MCIN/AEI/10.13039/501100011033/ and FEDER (“A way to build Europe”).

## Conflict of interest

The authors declare that the research was conducted in the absence of any commercial or financial relationships that could be construed as a potential conflict of interest.

## Publisher’s note

All claims expressed in this article are solely those of the authors and do not necessarily represent those of their affiliated organizations, or those of the publisher, the editors and the reviewers. Any product that may be evaluated in this article, or claim that may be made by its manufacturer, is not guaranteed or endorsed by the publisher.
